# Radiotherapy combined with anlotinib for refractory leiomyosarcoma: a case report and literature review

**DOI:** 10.3389/fonc.2025.1490941

**Published:** 2025-07-21

**Authors:** Ziming Li, Xiuchun Yu, Ming Xu

**Affiliations:** Department of Orthopaedics, 960th Hospital of the PLA, Jinan, Shandong, China

**Keywords:** radiotherapy, anlotinib, leiomyosarcoma (LMS), literature review, case report

## Abstract

Refractory leiomyosarcoma (LMS) is characterized by notoriously high recurrence rates and poses significant surgical challenges due to its anatomical complexity and invasive growth patterns. When complete surgical resection proves unattainable, radiotherapy has emerged as a cornerstone therapeutic modality, with emerging evidence suggesting synergistic effects when combined with novel chemotherapeutic agents. This study presents an illustrative case of advanced popliteal fossa LMS managed through precision radiotherapy combined with anlotinib, a multi-target tyrosine kinase inhibitor, which achieved sustained local tumor control and progression-free survival over 18 months of follow-up. Notably, the comprehensive management strategy for treatment-related complications, particularly radiation-induced dermatitis and hematological toxicity, demonstrated clinically validated mitigation approaches through phased dose adjustment and supportive care protocols. The therapeutic paradigm described herein provides valuable insights for optimizing multimodal management of refractory soft tissue sarcomas, highlighting the potential of targeted therapy-radiotherapy combinations while emphasizing the critical importance of proactive complication surveillance in contemporary oncological practice.

## 1 Introduction

LMS is a type of soft tissue malignant tumor that originates from smooth muscle tissue (1–3), accounting for 5% to 10% of all soft tissue sarcomas. It typically arises from the smooth muscle layers of the intestinal wall, vascular smooth muscle, or mucosal muscle, with common sites of occurrence including the uterus, retroperitoneum, skin, blood vessels, and bones. Clinically, LMS may present as a retroperitoneal mass accompanied by pain, along with secondary symptoms such as constipation, urinary dysfunction, anal discomfort, sacrococcygeal or lower limb pain, and generalized fatigue due to tumor-induced compression of adjacent tissues. Hematogenous spread is the primary metastatic pathway of LMS. While this malignancy predominantly affects middle-aged and elderly individuals, rare cases have been reported in pediatric and adolescent populations. Refractory LMS pertains to those that do not respond effectively to conventional treatments (such as surgery, chemotherapy, and radiation) or that relapse or progress rapidly after treatment. The treatment of refractory LMS is frequently intricate and demands a comprehensive contemplation of multiple therapeutic approaches. 1. Surgical resection: For early detection and when the tumor location and size permit, surgical resection is the preferred treatment. However, in cases of refractory LMS, surgical removal may become arduous or unfeasible as the tumor might have invaded the surrounding tissue or have distant metastases. 2. Chemotherapy: Chemotherapy is a common treatment modality for LMS, particularly for tumors that are not removable surgically (4). Commonly utilized chemotherapy drugs include doxorubicin, gemcitabine, docetaxel, etc. For refractory LMS, different chemotherapy regimens or combinations of drugs might need to be attempted. 3. Radiotherapy: Radiotherapy can be employed as an adjuvant treatment before and after surgery to assist in reducing the risk of recurrence and controlling local symptoms (5). But for tumors that are already insensitive to chemotherapy, the effect of radiation might be limited. 4. Targeted therapy: Targeting specific molecular targets of LMS, such as vascular endothelial growth factor receptor (VEGFR), platelet-derived growth factor receptor (PDGFR), etc (6–8), the utilization of corresponding targeted drugs (such as evacizumab, arotinib, pazopanib, etc) may aid in controlling tumor growth (7, 9, 10). These drugs often have minor side effects, but not all patients will respond to targeted therapy. 5. Immunotherapy: Immune checkpoint inhibitors (such as PD-1/PD-L1 inhibitors) have demonstrated efficacy in certain types of tumors, including some sarcomas (11, 12). Immunotherapy may be an exploratory treatment option for refractory LMS, especially when other treatments have failed. 6. Individualized treatment: Based on a patient’s genetic test results, specific genetic mutations may be identified that could guide the usage of specific targeted drugs. For instance, some patients with LMS with specific genetic mutations may benefit from specific targeted therapies. 7. Clinical trials: Participating in clinical trials may be a means to gain access to the latest treatments, especially for patients who have already attempted standard treatments but have not responded favourably. When treating refractory LMS, the collaboration of a multidisciplinary team (MDT) is critical to ensure that patients have access to the comprehensive treatment plan best suited to their condition. Patients should communicate closely with their healthcare team to understand the pros and cons of various treatment options and make treatment decisions guided by their doctor. Radiation therapy (referred to as radiotherapy), surgery and drugs are the three major treatment methods for malignant tumors. In the treatment of multiple malignancies, radiotherapy combined with targeted therapy can extend the time of local tumor control and reduce mortality without increasing the toxicity associated with radiotherapy. Radiotherapy mainly uses radiation to kill tumor cells, while targeted therapy inhibits tumor growth by targeting specific molecular targets on the surface of tumor cells. They act together on tumor cells through different mechanisms and exert synergistic anti-tumor effects. Currently, surgery is the only possible cure for LMS, but clinicians often lack experience in treating LMS that is difficult to remove surgically. This paper retrospectively analyzed the prognosis of a patient with refractory popliteal LMS treated by radiotherapy combined with anlotinib in our hospital, and combined with the literature, provided a reference scheme for the clinical treatment of refractory LMS.

## 2 Case description

**Figure 1:**
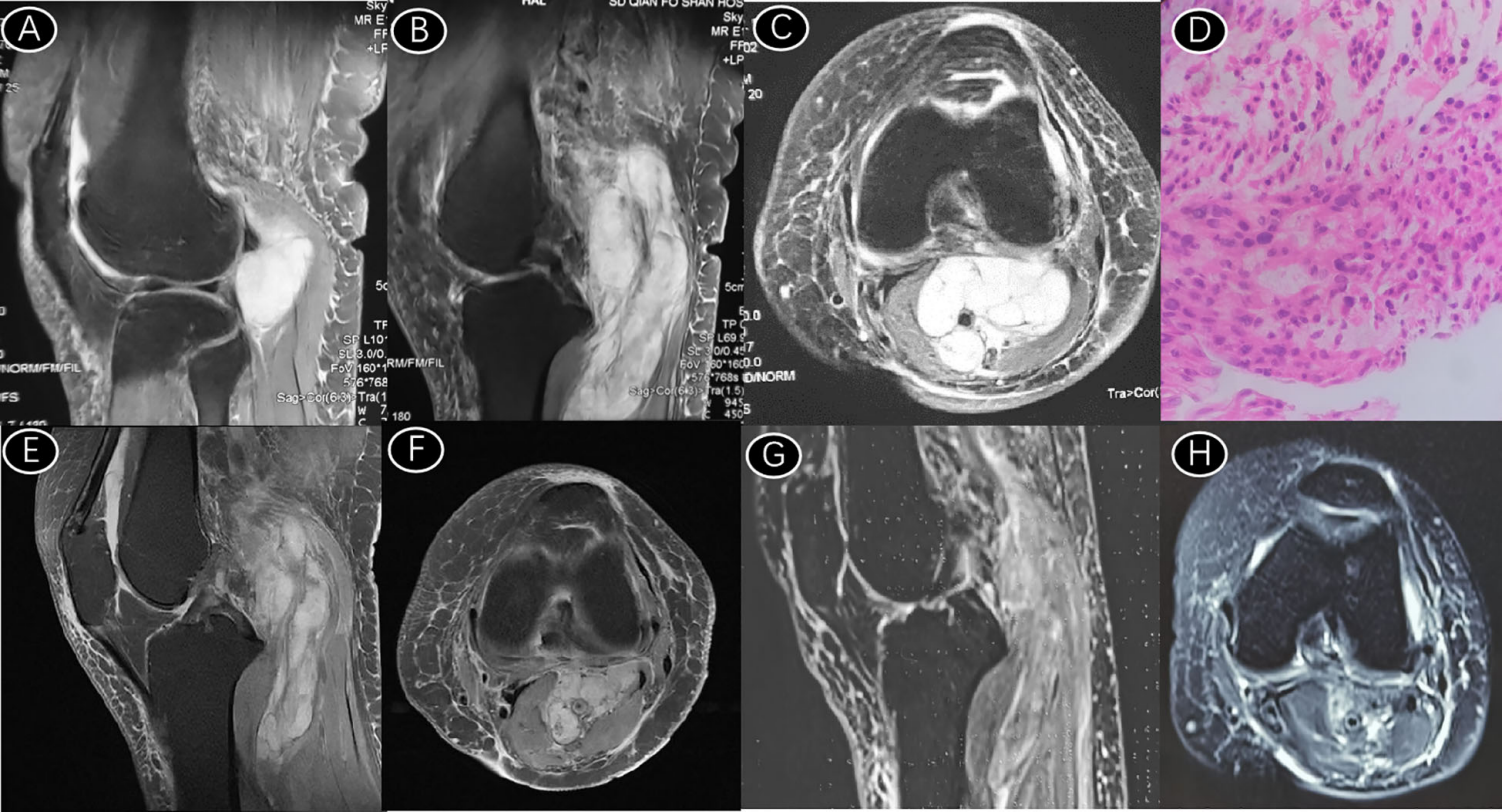
**(A–C)** Long T1 and T2 signal shadow in the left popliteal fossa, wrapping popliteal artery, vein and tibial nerve; **(D)** Puncture pathology showed malignant spindle cell mesenchymal tumor (left popliteal fossa), immunohistochemical staining results were CD99 (+), Desmin (+), Calpon (+), CD68 (scattered +), EMA (focally weak +), CD34 (-), CK (-), P53 (clone: DO-7) (-), SOX-10 (-), Mayglobin (-), MyoD1 (-), MUC4 (-), Ki-67 (clone: 7B11) (about 40%+). Combined with immunohistochemical staining, the results tended to be LMS. **(E, F)** After 10 fractions of intensity-modulated radiotherapy and 3 fractions of stereotactic radiotherapy, the tumor boundary was significantly clearer than before. **(G, H)** Three months after oral administration of anlotinib capsules, further reduction in tumor volume and clear boundaries were observed.

The 84-year-old female patient was admitted to the hospital on May 9, 2022, due to “pain and swelling in the left lower limb for 9 months and a mass in the left popliteal fossa for 1 month”. The patient had a history of over 60 years of Meniere’s syndrome, more than 40 years of hypertension, and 16 years of bronchiectasis. The physical examination indicated lameness. The left popliteal fossa could touch a mass approximately 7*8 cm in size, with a tough texture, indistinct boundary, slightly elevated skin temperature, poor mobility, mild tenderness, normal local skin color, no skin rupture or superficial venous irritation, slightly swollen left calf and left foot, and the left knee joint mobility ranged from 0 to 100 degrees. The skin sensation, muscle strength, and muscle tension of both lower limbs were normal, the left dorsal foot artery pulsated well, the physiological reflex was present, and the pathological reflex was not elicited. Imaging examination revealed irregular mass abnormal signals behind the left knee joint. T1W1 showed a high signal, T2W1 showed a high signal, and lipomatosis showed a high signal, surrounding the popliteal nerve, vein, and tibial nerve (see **Figures 1A–C**). The patient underwent tumor biopsy in our hospital on May 10, 2022, and the pathology disclosed LMS (see **Figure 1D**), tends to belong to high-grade LMS. The patient was treated with radiotherapy. The specific radiotherapy scheme was intensity-modulated radiotherapy for popliteal lesions at 400 cGy * 10 times + stereotactic radiotherapy with an additional dose of 700 cGy * 3 times and a cumulative dose of 6100 cGy. After radiotherapy, antrotinib hydrochloride capsule 12mg was administered orally, 1 capsule per day, before breakfast, for 2 consecutive weeks, and the drug was withheld for 1 week. A total of 3 weeks constituted a treatment cycle. Successive reexaminations demonstrated that the tumor volume was effectively controlled and decreased (see **Figure 1**). The patient’s pain were significantly alleviated, and the flexion and extension activities of the knee joint were significantly improved compared with those before treatment. The popliteal mass was markedly reduced and not palpable to palpation, the local skin temperature was normal, and the swelling of the lower limbs was reduced, but there was still a mild nocturnal pain. Pathological findings of the patient’s tumor showed positivity for Desmin/Calponin, confirming smooth muscle differentiation. Such tumors are often associated with VEGFR2 overexpression (though not directly tested in this case, the positivity rate in LMS is approximately 60-70%). Leiomyosarcoma (LMS) has been demonstrated to be sensitive to anti-angiogenic drugs. Anlotinib, as a multi-target tyrosine kinase inhibitor, simultaneously inhibits VEGFR2/3, PDGFR-α/β, and FGFR1-4. The high Ki-67 expression (40%) suggests active tumor proliferation, necessitating intensified anti-angiogenic therapy to disrupt nutrient supply. Anlotinib can reduce tumor angiogenesis and diminish blood supply to the tumor. CD34 negativity rules out VEGFR-low-sensitive subtypes such as solitary fibrous tumor.

In this case, as the popliteal fossa tumor was close to the popliteal artery, vein, and nerve, it was challenging to be completely removed through surgery. Moreover, the patient was elderly and had multiple underlying diseases, thus conventional surgical resection was not selected as the treatment plan. After detailed communication with the radiotherapy physician and the patient’s family members, radiotherapy combined with oral targeted drug therapy was determined, and the final clinical effect also proved that this was an alternative treatment plan worth considering.

**Figure 2:**
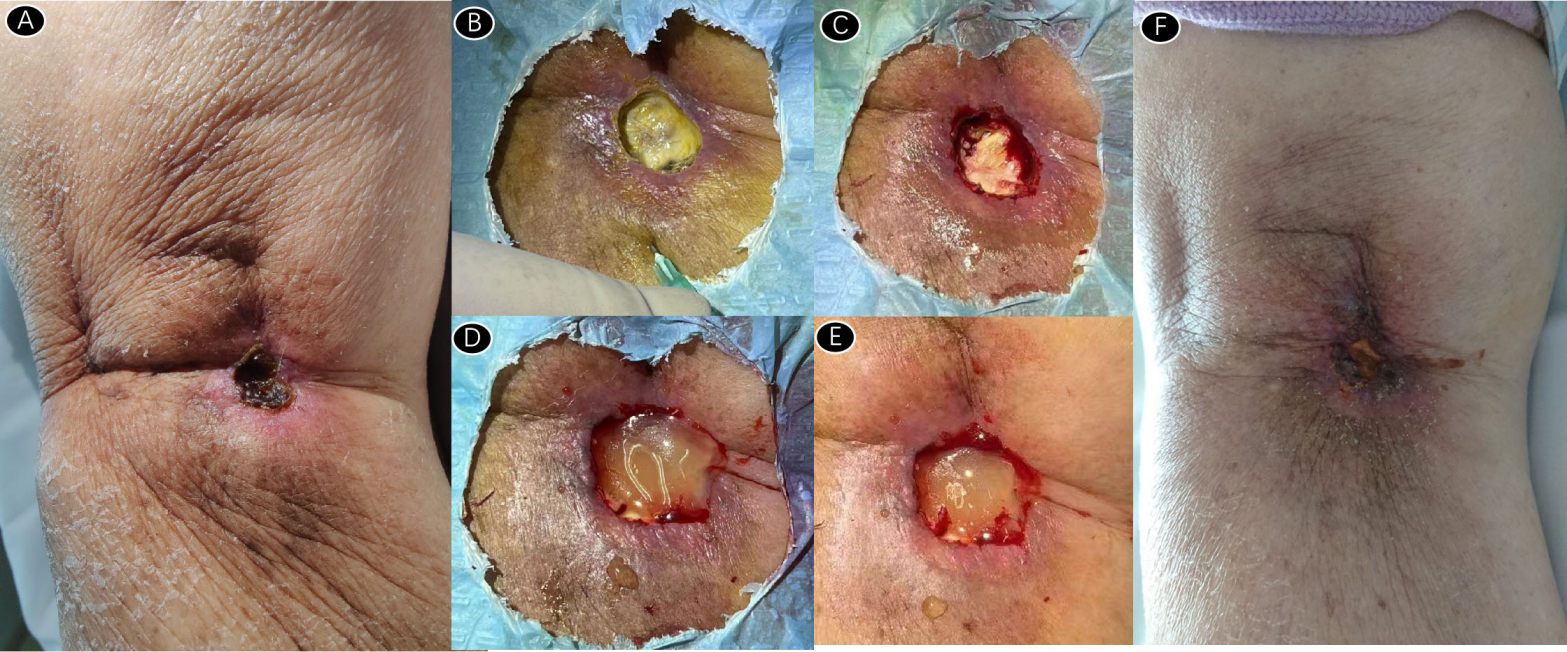
**(A)** skin ulcer with a size of about 2×2cm appeared in the popliteal fossa. **(B–E)** after 4 times of debridement +PRP injection; **(F)** After 4 treatments, the skin ulcer surface healed well.

After 13 times of radiotherapy, the patient developed skin ulceration with exudation in the popliteal fossa. After 4 times of debridement and platelet-rich plasma (PRP) treatment, the wound healed gradually. (See **Figure 2**) During debridement, the wound secretions were scraped clean until fresh bleeding, and then PRP gel supplemented with thrombin was applied. Thrombin can be used to coagulate PRP into a gel, which can not only bind tissue defects, but also prevent the loss of platelets, so that platelets can secrete growth factors for a long time and maintain a high concentration of growth factors. PRP contains a high concentration of activated growth factors to accelerate wound healing. It has been applied to the treatment of trauma injuries and ulcers at present (13). Since the proportion of each growth factor is similar to the normal physiological concentration *in vivo*, it can make the best synergistic effect between each growth factor. After applying PRP for 4 times within 1 month, the wound gradually shrank and dried to form blood crust. At the end of the follow-up of this study, the popliteal fossa wound healed well without obvious pain and no signs of tumor recurrence.

## 3 Discussion

### 3.1 Progress in the treatment of popliteal LMS

LMS is a typical malignant tumor, and its biological behavior depends on the location of its occurrence. It is challenging to completely remove it when it invades blood vessels and nerves (14). The lesion of the patient was located in the popliteal fossa, a diamond-shaped space between the lower leg and the thigh at the posterior side of the knee joint. The popliteal fossa is a special location with complex anatomy and is a common site for soft tissue tumors.

In clinical practice, popliteal LMS is typically treated with extensive surgical resection. However, the popliteal artery, vein, and nerve are distributed in the popliteal fossa. When the surgical margin is close or positive, radiotherapy serves as an important auxiliary means for local control (2).

Anlotinib can inhibit multiple targets, such as vascular endothelial growth factor receptor, platelet-derived growth factor receptor, fibroblast growth factor receptor, C-Kit, and RET genes (15, 16), and has been extensively utilized in the clinical treatment of lung cancer, soft tissue sarcoma, thyroid cancer, and other malignant tumors in China (17, 18).

### 3.2 Radiotherapy for popliteal soft tissue tumors

Radiotherapy, or radiation therapy, is a modality for treating malignant tumors by employing high-energy rays. This approach, which eliminates cancer cells through the action of ionizing radiation, is one of the three principal means of cancer treatment, along with surgery and chemotherapy. The principle of radiotherapy is to utilize radiation (such as X-rays, gamma rays, electron beams, proton beams, etc.) to damage the DNA of cancer cells, thereby suppressing their growth and reproduction.

The role and status of radiotherapy in tumor treatment have become increasingly prominent. Approximately 70% of cancer patients require radiotherapy during treatment, and approximately 40% of cancers can be cured by radiotherapy. Radiotherapy can be employed as an independent treatment or in combination with other treatment modalities, such as surgery and chemotherapy, to enhance the therapeutic effect (19–21).

The main forms of radiotherapy encompass: 1. Long-course external beam radiotherapy: once a day, five times a week, with a total treatment time of approximately 5–7 weeks, and each dose being 1.8-2.0 Gy. It is suitable for large tumors and tumors that are moderately or more sensitive to radiation. 2. Short-course external beam radiotherapy: once a day, 1–10 treatments, with the treatment completed within two weeks, and each treatment dose above 5.0 Gy. It is suitable for tumors less than 3cm in diameter and tumors that are not sensitive to radiation. 3. Brachytherapy: The radioactive source is placed within the patient’s body and treated in proximity to the tumor target area. It includes high-dose-rate “afterloading” therapy and iodine-125 seed implantation.

The selection of radiotherapy depends on the type, size, and location of the tumor, as well as the overall condition of the patient. With the advancement of technology, radiotherapy equipment and methods have been continuously enhanced, such as three-dimensional conformal radiotherapy (3D-CRT), intensity modulated radiotherapy (IMRT), stereotactic body radiotherapy (SBRT), etc. These technologies have improved the accuracy of radiotherapy and reduced the damage to surrounding normal tissues.

The treatment will be customized according to the patient’s specific condition to minimize side effects and enhance the quality of life. In some cases, radiotherapy can also be utilized to alleviate pain and other symptoms and improve the quality of life of patients with advanced cancer. Although radiotherapy is an effective treatment modality, it may also induce certain side effects, such as skin reactions, radiation bone injury (22, 23). Radiation-induced complications need to be seriously considered in clinical practice, among which radiation-induced dermatitis is the most common complication (24, 25). Due to the presence of skin wrinkles, popliteal soft tissue tumors are prone to radiation-induced dermatitis during radiotherapy. Drugs, such as plant extracts (including aloe vera gel and plant oils), vitamins (including vitamin C, vitamin B1, and vitamin E, etc.), creams (hormone creams), recombinant human epidermal growth factor, and traditional Chinese medicine, are important approaches for the prevention and treatment of radiation dermatitis.

### 3.3 Research progress of anlotinib in the treatment of malignant tumors

Anlotinib is a novel small-molecule multi-target tyrosine kinase inhibitor (TKI). It exerts anti-tumor effects primarily by inhibiting VEGFR, PDGFR, and fibroblastic growth factor receptor (FGFR). Anlotinib can effectively inhibit tumor angiogenesis, thereby suppressing tumor growth and metastasis.

Anlotinib has demonstrated promising efficacy in the treatment of various malignancies,including but not limited to non-small cell lung cancer (NSCLC), small cell lung cancer (SCLC), medullary thyroid carcinoma, and soft tissue sarcoma. In China, anlotinib has been approved for the treatment of advanced NSCLC, SCLC, medullary thyroid carcinoma and soft tissue sarcoma. In addition, it has been widely studied in the treatment of hepatocellular carcinoma, renal cancer and gastrointestinal cancer.

In the treatment of non-small cell lung cancer, anlotinib can be used as a single agent or in combination with other drugs such as chemotherapy, anti-angiogenesis inhibitors, and immune checkpoint inhibitors. Clinical studies have shown that anlotinib monotherapy can significantly improve the progression-free survival (PFS) and overall survival (OS) of patients with advanced NSCLC. In combination therapy, the combination of anlotinib with other drugs has also shown good efficacy and safety.

However, anlotinib treatment is also accompanied by some adverse reactions, including hypertension, fatigue, hand-foot syndrome, diarrhea, anorexia, proteinuria and so on. Physicians and patients need to closely monitor these adverse effects and take corresponding management measures, such as dose adjustment and symptomatic treatment, to ensure patient safety and continuity of treatment.

### 3.4 Radiotherapy combined with Anlotinib for the Treatment of Malignant Tumors

Anlotinib combined with SBRT in the treatment of Pulmonary Carcinosarcoma showed significant tumor improvement with partial response (PR) (26). In the treatment of NSCLC, anlotinib may, in some cases, lead to better outcomes when used in combination with radiotherapy. For example, in patients with NSCLC with brain metastases (BMs), anlotinib combined with radiotherapy may help extend PFS and OS while controlling the growth of BMs. The LAURA study was the first phase III clinical trial to explore targeted therapy for Stage III EGFR-Mutated NSCLC. The median PFS after consolidation chemoradiotherapy with osimertinib was 39.1 months, compared with 5.6 months in the chemoradiotherapy group, which significantly improved PFS (27).

Limitations: At present, there is a lack of large-scale clinical reports on radiotherapy combined with anlotinib in the treatment of malignant tumors.

## 4 Conclusions

In conclusion, leiomyosarcomas of the popliteal artery are rare neoplasias belonging to the group of soft tissue sarcomas of the limbs of vascular origin. The patient received radiotherapy combined with anlotinib treatment, and the tumor was effectively controlled and the complications were satisfactory, providing a new treatment opportunity for refractory LMS. However, in the future, more basic and clinical studies are expected to explore the efficacy and safety of radiotherapy combined with anlotinib, in order to obtain good tumor control and translate into survival benefits.

## Data Availability

The raw data supporting the conclusions of this article will be made available by the authors, without undue reservation.
